# 

**Published:** 1844-01

**Authors:** 


### WESTERN JOURNAL

OF

### MEDICINE AND SURGERY.

### EDITORS.

DANIEL DRAKE, M. D.,

PROFESSOR OF PATHOLOGICAL ANATOMY AND CLINICAL MEDICINE IN THE MEDICAL INSTITUTE OF LOUISVILLE:

LUNSFORD P. YANDELL, M. D.,

PROFESSOR OF CHEMISTRY AND PHARMACY IN THE SAME:

THOMAS W. COLESCOTT, M. D.

### BOOKS RECEIVED.

We have received from the publishers, Lea & Blanchard, the following works for notice in our Journal: Williams' Principles of Pathology, Harrison on the Nervous System, and Watson's Practice of Physic. They will be reviewed as soon as time and space can be commanded. We have also received from the author, Dr. Edward Jarvis, of Dorchester, Massachusetts, an interesting paper on "Insanity of the Colored Population of the Free States," in which some singular errors in the sixth census of the United States are brought to light. We shall notice the subject in our next number.

---

### WESTERN MEDICAL SCHOOLS.

Kemper College, at Saint Louis, is said to have 90 students in attendance on the medical lectures, this winter. At Cincinnati there are reported to be about 180. A gentleman residing at Lexington, writes to a friend in this city, that the number at that school is 212. The number at the Louisville Medical Institute is 243. We have not heard from either the new school at St. Louis, or the New Orleans school.

### TO OUR SUBSCRIBERS.

We must again remind our subscribers of their arrearages to us, and *urge* them to remit their respective dues. Our expenses for the last and present volume have been more than usually heavy, whilst the receipts, we are sorry to say, have by no means augmented in like proportion. Those who owe for this Journal from the commencement, will not, we trust, suffer us to ask them again for the amounts due us. We hope, moreover, that *all* our subscribers, whether old or recent, will feel it incumbent to remit immediately.

Thus far, we have not collected enough to defray the cost of the Journal, but we are determined to sustain it. We design commencing the ninth volume, with new and improved materials—will not the subscribers encourage and sustain us by remitting forthwith by mail, at our risk, franked by the Postmaster?

*October*, 1843. PRENTICE & WEISSINGER.

---

### RECEIPTS FOR MEDICAL JOURNAL SINCE OUR LAST.

| | |
|---|---|
| G. G. Boyle, Providence, Ky., - - - - | $10 00 |
| W. E. Murphy, Decatur, Ala. - - - - | 5 00 |
| W. Long, New Maysville, Ia., - - - - | 5 00 |
| W. Bell, Bellbrook, Ohio, - - - - | 10 00 |
| H. Hard, Plymouth, Ia. - - - - - | 10 00 |
| J. Slavens, Portland Mills, Ia. - - - - | 10 00 |
| T. S, Head, Meadville, Miss., - - - - | 10 00 |
| E. Foster, Louisville, Miss., - - - - | 5 00 |
| H. M. Brown, Newtown, Ohio, - - - - | 10 00 |
| V. T. West, Union, Ia., - - - - | 5 00 |
| Dr. L. Jackson, Columbus, Ia., - - - - | 3 00 |
| A. Moore, Raleigh, Tenn., - - - - | 5 00 |
| F. C. Ewing, Spring Hill, Tenn., - - - | 5 00 |
| J. Lea, Auburn, Mo., - - - - - | 10 00 |
| Dr. C. White, Paoli, Ia., - - - - | 5 00 |
| W. H. Ruffin. Warsaw, Mo. - - - - | 5 00 |
| E. L. Miner, Lethopolis, Ala., - - - - | 10 00 |
| J. L. Williams, Camden, Tenn., - - - - | 10 00 |
| L. Loop, Thorntown, Ia., - - - - | 10 00 |
| Dr. Barton, Waterford, Pa., - - - - | 10 00 |
| Dr. Speers, Lynchburg, Ohio, - - - - | 8 00 |
| Dr. J. Hardin, Greensburg, Ky., - - - - | 5 60 |
| H. Burritt, Gilead, Ohio, - - - - - | 10 00 |
| H. Hard, Plymouth, Ia., - - - - - | 1 00 |
| Dr. B. F. Todd, Middletown, Mo., - - . | 15 00 |
| J. Roberts, Montezuma, Ia., - - - - | 5 00 |
| W. Blackstone, Athens, Ohio, - - - - | 5 00 |
| S. M. Linton, Columbus, Ia., - - - - | 5 00 |
| J. Barnes, Decatur, Ill., - - - - - | 2 00 |
| Dr. Murray, Mouth of White River, Ark., - - | 5 00 |
| J. M. Morris, Somerset, Ohio, - . - - | 5 00 |
| B. B. Smith, Shreveport, La., - - - - | 10 00 |

The Western Journal of Medicine and Surgery is published monthly by the undersigned, at the corner of Main and Fifth streets, Louisville, at $5 per annum, payable in advance. Each number contains 96 pages, making two volumes in the year of more than 550 pages.

Contributions to its pages by the Physicians of the Valley of the Mississippi, addressed to the Editors, are respectfully solicited.

Letters on business, to be addressed, postage paid, to the publishers.

☞Postmasters, by the regulations of the Postoffice Department, will frank letters containing subscription money, and all remittances so franked are at the risk of the publishers.

*January*, 1844. PRENTICE & WEISSINGER.

